# Insights into the Crystal Structure and Magnetodielectric Properties of High-Energy Ball Milled Sr Substituted LaFeO_3_

**DOI:** 10.3390/ma18133014

**Published:** 2025-06-25

**Authors:** Julio C. Aguirre-Espinosa, Félix Sánchez-De Jesús, Claudia A. Cortés-Escobedo, Ana M. Bolarín-Miró

**Affiliations:** 1 Área Académica de Ciencias de la Tierra y Materiales, Universidad Autónoma del Estado de Hidalgo, Mineral de la Reforma, Hidalgo 42184, Mexico; ag133712@uaeh.edu.mx (J.C.A.-E.); fsanchez@uaeh.edu.mx (F.S.-D.J.); 2 Centro de Investigación e Innovación Tecnológica, Instituto Politécnico Nacional, Ciudad de México 02250, Mexico; ccortese@ipn.mx

**Keywords:** lanthanum ferrite, LaFeO_3_, magnetodielectric coupling, strontium doping, multiferroic, high-energy ball milling

## Abstract

The effect of strontium substitution on the crystal tructure, as well as the magnetic, and electrical properties of lanthanum ferrite (LaFeO_3_) synthesized by high-energy ball milling, is studied, with an emphasis on magnetodielectric coupling. X-ray diffraction (XRD) confirmed the successful synthesis of orthorhombic La_1−x_Sr_x_FeO_3_ for doping levels up to 0.2 mol. At 0.3 mol Sr^2+^, two phases appear: La_0.6_Sr_0.4_FeO_2.976_ and La_0.8_Sr_1.2_FeO_3.714_, the latter being metastable. This phase vanishes at 0.5 mol. The Fourier Transform Infrared Spectroscopy (FT-IR) and Scanning Electron Microscopy coupled with Energy Dispersive X-ray Spectroscopy (SEM-EDS) analysis confirmed these results using a vibrating sample magnetometer (VSM), whose measurements show ferromagnetism at 0.1 and 0.3 mol Sr^2+^, attributed to crystal distortion, magnetic spin rearrangement, and as consequence, modifications in the double-exchange interactions. Dielectric tests reveal that higher Sr^2+^ concentrations lead to increased relative permittivity, dielectric losses, and conductivity, linked to oxygen vacancy formation. This study demonstrates a room-temperature magnetodielectric coupling of 32% in Sr-doped lanthanum ferrite, highlighting its potential for technological applications.

## 1. Introduction

Multiferroic materials with magnetoelectric (ME) coupling exhibit the simultaneous presence of ferromagnetism (FM) or antiferromagnetism (AFM) and ferroelectricity (FE). The coupling of ferroelectric and ferromagnetic polarization opens up the possibility of designing new devices, such as sensitive magnetic field sensors, non-volatile memories, and logic elements, as well as energy harvesting devices, among others [1]. However, bismuth ferrite and lanthanum ferrite are the only single-phase ME multiferroic compounds at room temperature known to date [2]. Therefore, the design for a material that possesses both electric and magnetic polarization at room temperature is still in progress.

Rare-earth orthoferrites are of interest due to their electric, magnetic, and optical properties, as a consequence of their crystal structure with a distorted perovskite-type spatial organization of the ABO_3_ type. In this structure, the A-cations are located in the center of the unit cell, while the B-cations are located at the corners, surrounded by a distorted octahedron composed of oxygen anions from the ABO_3_ structure. The crystal structure offers the possibility of making relatively simple modifications capable of changing their electrical and magnetic properties. These modifications can be achieved through distortions by synthesis methods and doping at various positions of the crystal structure. The magnitude of this distortion depends on the characteristics of the doped ions and the location [3].

Among the orthoferrites, LaFeO_3_ is highlighted since shows multiferroic properties, in particular, LaFeO_3_ presents: (i) ferroelectric ordering induced by the strong local electric field generated through lattice distortion caused by the 3d electrons of the transition metal ion, (ii) antiferromagnetic insulating behavior with a high Néel temperature ~740 K, and (iii) mixed ion and electron-hole conductivity over a wide range of pressure and temperature [4]. Depending on the oxygen partial pressures, it can exhibit either p-type (electron vacancies) or n-type (formation of anion, and/or cation reduction) conductivity. In addition, by doping oxygen-conducting ceramics with multivalent cations, it is possible to achieve the coexistence of ionic and electronic conductivity [5,6]. In this sense, LaFeO_3_ has been studied in depth [7] and has been reported as multiferroic at room temperature [8]. In addition to these characteristics, LaFeO_3_ exhibits a wide range of functional properties that further enhance its technological relevance. It shows significant catalytic activity for redox reactions such as CO and hydrocarbon oxidation, NO_x_ reduction, and water splitting, making it useful in gas sensors and environmental remediation applications [9]. Its narrow bandgap (~2.1–2.3 eV) also enables potential use in photocatalysis and photoelectrochemical cells [10]. Furthermore, LaFeO_3_ is highly sensitive to structural changes induced by external stimuli, such as strain, temperature, and doping, which can substantially modulate its electrical, magnetic, and optical responses [11]. This multifunctional tunability makes it a strong candidate for integrated electronic systems where synergistic coupling between electric, magnetic, and optical functions is desired.

Although LaFeO_3_ exhibits interesting properties, it shows antiferromagnetic order, low electric polarization, and weak coupling between electric and magnetic orderings at room temperature, which limits its technological applications. In addition, the magnetoelectric coupling in the pure LaFeO_3_ in bulk is limited.

To overcome these limitations, numerous investigations have been carried out focusing on the effect of doping on lanthanum and iron sites, and how redox behavior, transport properties, thermal stability, and lattice structure are influenced, and in this sense, the substitution of alkaline earth elements such as Sr, Ba or Ca, have shown a favorable effect on the aforementioned properties and characteristics [12,13].

Furthermore, Lataoui et al. [14] proposed that the substitution of La^3+^ by Sr^2+^ induces a decrease in the unit cell volume and changes in the lattice size. Similarly, the conductivity increases with the increase in the doping level. Additionally, Paiva et al. [15] reported that there is a transformation of the crystal system from orthorhombic to rhombohedral when the strontium content increases from 0.2 to 0.4 mol for the pellets that were sintered at 1423 K for 2 h. By carrying out an Ab initio study, Ritzmann et al. [16] demonstrated that both the volume of the unit cell and the Fe-O bond length decrease, while the Fe-O-Fe angles increase as the strontium content changes from 0 to 0.5 mol. Additionally, oxygen vacancies are created due to the reduction in Fe ions induced to maintain electroneutrality. On the other hand, the study of the La_x_Sr_1−x_FeO_3_ system shows that the iron valence state gradually shifts from Fe^3+^ to Fe^4+^ as the value of x increases, indicating an intermediate valence state for iron. Alternatively, it suggests that the trivalent state of iron remains stable while oxygen vacancies are generated in the lattice to maintain electroneutrality in the compound. The partial substitution of La^3+^ by Sr^2+^ facilitates the gradual structural transition from orthorhombic to cubic, accompanied by an increase in oxygen nonstoichiometry [17].

Regarding the magnetic properties, the notable increase in magnetization with the addition of Sr^2+^ could be ascribed to the generation of Fe^4+^ ions, which reinforce the ferromagnetic component by participating in the double-exchange (DE) interaction, Fe^4+^-O^2−^-Fe^3+^, alongside the creation of oxygen vacancies that disrupt the uncompensated surface spin. AC-conductivity measurements revealed a shift in the conduction mechanism from electronic to ionic as Sr^2+^ content increases, leading to enhanced conductivity [18]. It is congruent with Gowri et al. [19] that report that the possible cause for the induction of ferromagnetism when the strontium content ranges between 0.05 and 0.20 mol is the magnetic moment caused by the uncompensated spins of Fe^3+^ and La^3+^ ions, which increases remanence and coercivity. This occurs due to the presence of a higher quantity of Fe^3+^ ions and, consequently, uncompensated spins, which reinforce the antisymmetric exchange interaction between Fe^3+^ and La^3+^ ions. Additionally, Yang et al. [20] determined that the primary factors influencing the coercivity of the samples include: magnetic anisotropy, grain size, microstrain, stress, crystal symmetry, spin–orbit coupling effect, magnetic single domain size, impurities, and calcination temperature.

Based on the aforementioned, the substitution of lanthanum by strontium in LaFeO_3_ induces a crystal structure distortion resulting in the structural change in the ferrite and/or the formation of metastable phases, which depends on the synthesis method. Additionally, the charge imbalance caused by strontium leads to the generation of vacancies, which increases the conductivity with increasing dopant concentration. On the other hand, this imbalance promotes the generation of Fe^4+^, which in turn favors DE magnetic interactions and, consequently, induces ferromagnetism in lanthanum ferrite.

While various synthesis methods have been employed to prepare Sr-doped LaFeO_3_ such as sol–gel combustion [21], microwave-assisted techniques [22], and molten salt processes [23] between others, these typically offer good control over morphology and particle size but often involve high temperatures, costly precursors, or meticulous control of synthesis conditions, which can hinder scalability and reproducibility. In contrast, high-energy ball milling (HEBM) has emerged as a promising alternative due to its simplicity, low cost, and versatility. This technique facilitates the formation of materials with high crystallographic defect concentrations, microstrain, and potential non-equilibrium phases that can significantly modify structural, electrical, and magnetic properties. Despite its advantages, the application of HEBM to Sr-doped LaFeO_3_ remains underexplored. In this study, HEBM is employed to synthesize Sr-doped LaFeO_3_, aiming to assess its influence on structural distortion, oxygen vacancy generation, and magnetic interactions. Furthermore, a comparative analysis is presented not only with conventional synthesis routes, but also with other doping systems (e.g., Ca^2+^, Ba^2+^), to clarify how Sr-doping mechanisms specifically affect structure–property correlations and multiferroic behavior [24]

Despite the numerous studies related to the synthesis and characterization of lanthanum ferrite doped with different cations due to the great technological interest, there is a lack of information about the effect of HEBM on the crystal structure, magnetic, and electric properties of Sr^2+^ doped lanthanum ferrite. It is anticipated that the HEBM will facilitate the rapid formation of the doped lanthanum ferrite and induce new properties attributed to the formation of non-equilibrium phases and microstrain, typically obtained when this synthesis method is used. This could result in the possible induction of ferromagnetism in the G-antiferromagnetic LaFeO_3_, along with an improvement in the ferroelectric properties, due to the differences in ionic radii and magnetic moment, and possibly including the magnetodielectric coupling, thus expanding potential applications.

## 2. Materials and Methods

The synthesis method involved the HEBM technique, using high-purity oxides, La_2_O_3_, Fe_2_O_3_, and SrO, all from Sigma-Aldrich (St. Louis, MO, USA), with purities ranging from 99% to 99.99%, as precursors. The precursors were mixed in stoichiometric proportions, following reaction (1), with the variable x ranging from 0 to 0.5 with increments of ∆x = 0.1.

(1 − x) La_2_O_3_ + 2xSrO + Fe_2_O_3_ + (x/2) O_2_ → 2La_1−x_Sr_x_FeO_3_ (1)

Once the powders were weighed to achieve a total of 5 g of compound, they were deposited in cylindrical containers made of hardened steel with a volume of 60 cm^3^. The milling process took place in a high-energy mill (SPEX model 8000D, Metuchen, NJ, USA), using a ball-to-powder weight ratio of 10:1. The milling process lasted 5 h, divided into three 90 min cycles and one 30 min cycle, with 30 min pause periods between each cycle to avoid overheating of the powders. Milling was carried out at ambient pressure and temperature. After milling, the mechanically activated powders were subjected to a uniaxial compaction process using a hydraulic press (ENERPAC model PUJ1201B, Menomonee Falls, WI, USA) at a pressure of 1000 MPa, with the objective of obtaining pellets of 10 mm in diameter and 0.5 mm in height. Subsequently, a sintering process was carried out at 1473 K for 4 h using a tubular muffle furnace (Lindberg/Blue M model STF54459C, Riverside, MI, USA) in an air atmosphere at atmospheric pressure, ensuring a stable oxygen partial pressure throughout the treatment and minimizing the risk of oxygen loss. For dielectric testing, the specimens were coated on both flat sides with an Au-Pd alloy by means of a sputter deposition process.

Crystallographic characterization was performed in a 2-theta range from 20° to 50° with a step size of 0.01° using an X-ray diffractometer (Bruker D8 Advance, Karlsruhe, Germany) with Cu Kα radiation (λ = 1.541874 Å). The Rietveld refinement from diffraction patterns was conducted using the free software Material Analysis Using Diffraction (MAUD) version 2.9998. The FTIR technique was employed to identify the chemical bonds in the material using a spectrophotometer FT-IR (PerkinElmer model Frontier, Monmouth County, NJ, USA). The surface morphology of the sintered pellets was observed by scanning electron microscopy (SEM) (HITACHI model TM3030, Tokyo, Japan) to examine surface features such as grain boundaries and surface defects. The SEM is equipped with an Energy Dispersive Spectroscopy (EDS) (QUANTAX model 75, Ettlingen, Germany) for chemical analysis. To evaluate magnetic properties, a vibrating sample magnetometer (MicroSense model EV7, Lowell, MA, USA) was used, applying a maximum magnetic field of ±18 kOe. The dielectric properties were determined over a frequency range of 100 Hz to 1 MHz using an LCR meter (Hioki 3532-50, Nagano, Japan). Magnetodielectric evaluations were conducted using a custom-made sample holder placed inside the magnetometer coupled with the LCR meter, where the change in permittivity with respect to the magnetic field was determined in the range of −18 to 18 kOe.

A total of five samples of each composition were synthesized and characterized to ensure the reproducibility and consistency of the experimental results. In addition, the measurements were repeated three times to minimize experimental error and confirm the reliability of the data.

## 3. Results and Discussion

### 3.1. Crystal Structure

**Figure 1:**
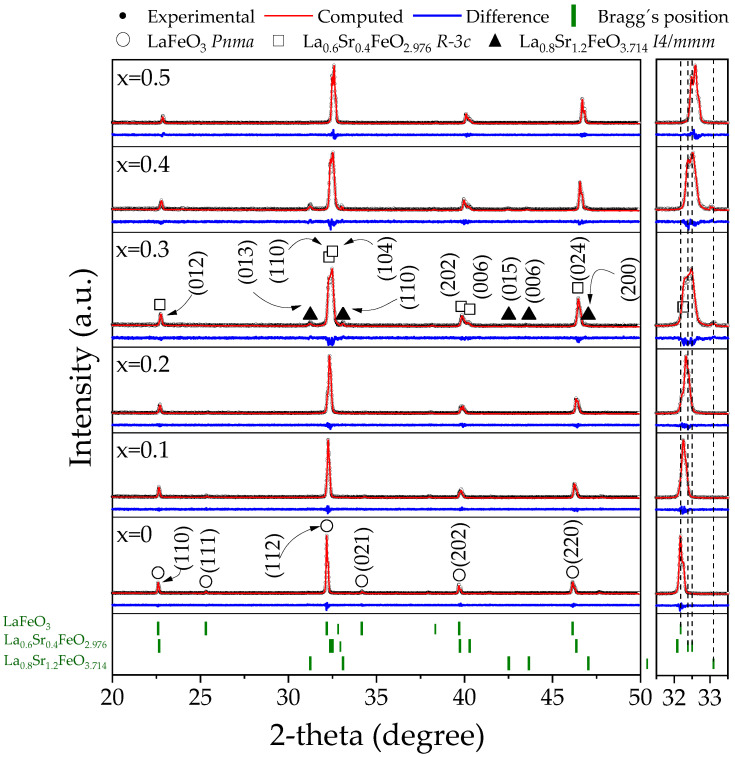
Rietveld analysis of XRD patterns of samples prepared by mixing La_2_O_3_, Fe_2_O_3_, and SrO powders at stoichiometric ratios, milling for 5 h, pressing, and sintering at 1473 K for 4 h for obtaining La_1−x_Sr_x_FeO_3_ with 0 ≤ x ≤ 0.5, Δx = 0.1.

X-ray diffraction (XRD) patterns and Rietveld refinement results of the La_1−x_Sr_x_FeO_3_ from sintered mixtures of La_2_O_3_, Fe_2_O_3,_ and SrO for obtaining La_1−x_Sr_x_FeO_3_ with 0 ≤ x ≤ 0.5, Δx = 0.1, are depicted in Figure 1. The analysis of the XRD patterns reveals the successful synthesis of single-crystalline LaFeO_3_ (x = 0) with orthorhombic structure (COD 96-156-1805, *Pnma*), assuring that the reaction (1) has been completed, where other phases were not detected. When strontium is added to lanthanum ferrite with a concentration of 0.1 mol a shift in the main peak is observed, located around 32° of 2-theta, towards higher angles, indicative of strontium incorporating into the crystalline structure of the ferrite, causing distortion in the unit cell attributed to the difference between the radii of lanthanum (136 pm) and strontium (144 pm) cations [25]. When the dopant content is increased up to 0.2 mol, the shift in the main diffraction peak is more evident, which is indicative that at this concentration strontium is still soluble in the lanthanum ferrite crystal arrangement.

When the dopant concentration is 0.3 mol of strontium, the intensity of the diffraction peaks corresponding to LaFeO_3_ diminishes, and the presence of new diffraction peaks is detected, attributed to the rhombohedral La_0.6_Sr_0.4_FeO_2.976_ (COD 96-153-3256, *R-3c*) and the tetragonal La_0.8_Sr_1.2_FeO_3.714_ (COD 96-153-2614, *I4/mmm*) [26]. The formation of additional phases at this composition is attributed to reaching the maximum solubility of strontium in the crystal structure of LaFeO_3_. When the strontium content is increased up to 0.4 mol, the diffraction peaks corresponding to the LaFeO_3_ have vanished, and the La_0.8_Sr_1.2_FeO_3.714_ phase prevails. Finally, as the strontium content is increased, the rhombohedral phase stabilizes as a single phase, confirming that the tetragonal phase acts as an intermediate stage that eventually transforms into the rhombohedral structure.

To complement the qualitative analysis and quantify the phases and cell parameters as a function of dopant concentration, Rietveld refinement was performed. The results obtained are presented in Table 1. The Rietveld refinement results confirm the successful synthesis of the doped LaFeO_3_ up to 0.2 mol of strontium, without secondary phases, as was previously indicated. The incorporation of Sr^2+^ into the LaFeO_3_ structure leads to a reduction in lattice parameters and crystallite size. This effect is attributed to a charge compensation mechanism, where the substitution of La^3+^ by Sr^2+^ induces the partial oxidation of Fe^3+^ to Fe^4+^ to maintain charge neutrality. Since the ionic radius of Fe^4+^ (58.5 pm) is smaller than that of Fe^3+^ (64.5 pm), the presence of Fe^4+^ results in a contraction of the crystalline structure, leading to the observed reduction in lattice parameters and crystallite size. Additionally, interactions between dopant and grain boundaries may lower the surface energy, stabilizing the surfaces and further contributing to the reduction in crystallite size [27]. The crystallite size values ranged from 57 to 189 nm, with a reduction in size observed up to the value of x = 0.2 for the LaFeO_3_ phase. For values from x = 0.3 to x = 0.5, where the La_0.6_Sr_0.4_FeO_2.976_ phase is present, an increase in crystallite size is observed. These values are related to the different strontium concentrations that modify the lattice parameters and the formation of new phases.

As previously mentioned, for strontium content of 0.3 mol, the coexistence of three phases, LaFeO_3_, La_0.6_Sr_0.4_FeO_2.976_, and La_0.8_Sr_1.2_FeO_3.714_, with proportions of 22.65%, 71.35%, and 5.99%, respectively, is quantified. However, when the strontium content reaches 0.4 mol, the orthorhombic ferrite phase disappears, leaving only La_0.6_Sr_0.4_FeO_2.976_ and La_0.8_Sr_1.2_FeO_3.714_. Finally, the highest doped ferrite studied, 0.5 mol strontium, presents a single rhombohedral phase, which confirms the metastable character of the La_0.8_Sr_1.2_FeO_3.714_. The goodness of fit values, χ^2^, and R_wp_, lower than 2 and 20, respectively, indicate a good fit of the performed Rietveld refinement.

### 3.2. FT-IR Analysis

**Figure 2:**
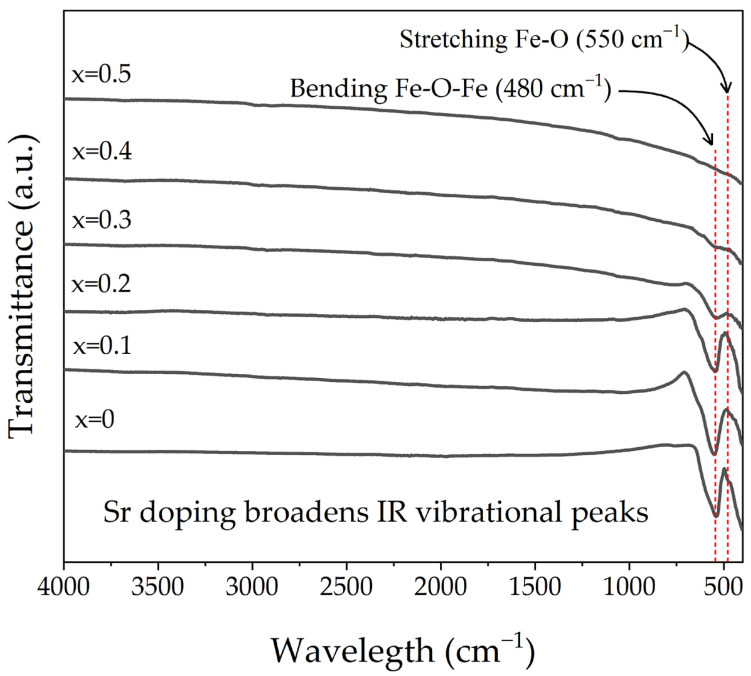
FT-IR spectra of samples prepared by mixing La_2_O_3_, Fe_2_O_3,_ and SrO powders at stoichiometric ratios, milling for 5 h, pressing, and sintering at 1473 K for 4 h for obtaining La_1−x_Sr_x_FeO_3_ with 0 ≤ x ≤ 0.5, Δx = 0.1.

Figure 2 shows the infrared (FT-IR) spectra of sintered pellets of La_1−x_Sr_x_FeO_3_ with strontium doping levels ranging from x = 0 to x = 0.5, recorded in the 4000–400 cm^−1^ region. For the undoped sample (x = 0), a sharp and intense band centered on 550 cm^−1^ is observed, which corresponds to the Fe-O stretching mode and a band around 480 cm^−1^ may be attributed to O-Fe-O deformation vibration characteristic of the orthorhombic perovskite structure [28]. The absence of additional bands confirms the presence of a single, pure phase in the samples with x = 0.1 and x = 0.2, also indicated by the XRD pattern. The main band remains near 550 cm^−1^ but becomes slightly broader and less intense, suggesting a progressive distortion of the crystal lattice due to the increasing Sr^2+^ content. This behavior is likely associated with the substitution of La^3+^ by Sr^2+^, which induces structural strain and oxygen vacancies as part of the charge compensation mechanism.

For x = 0.3 and x = 0.4, the main band in the FT-IR spectra, becomes significantly broader and shifts toward lower frequencies, indicating a more pronounced distortion of the Fe-O environment caused by the coexistence of multiple crystalline phases (orthorhombic, tetragonal, and rhombohedral), leading to increased structural complexity and reduced symmetry. Finally, in the x = 0.5 sample, the main Fe-O band is almost completely suppressed, which suggests a high degree of Sr^2+^ incorporation into the perovskite lattice and the stabilization of a rhombohedral phase with substantial lattice distortion. The decrease and eventual disappearance of the bands associated with the Fe-O stretching mode in samples with higher Sr content indicate that the incorporation of Sr into LaFeO_3_ introduces notable structural distortions within the crystalline framework. This modification promotes the formation of new Sr–O interactions, whose vibrations occur at lower frequencies, consistent with observations in other A-site doped perovskite systems [29].

### 3.3. Surface Morphology Analysis

**Figure 3:**
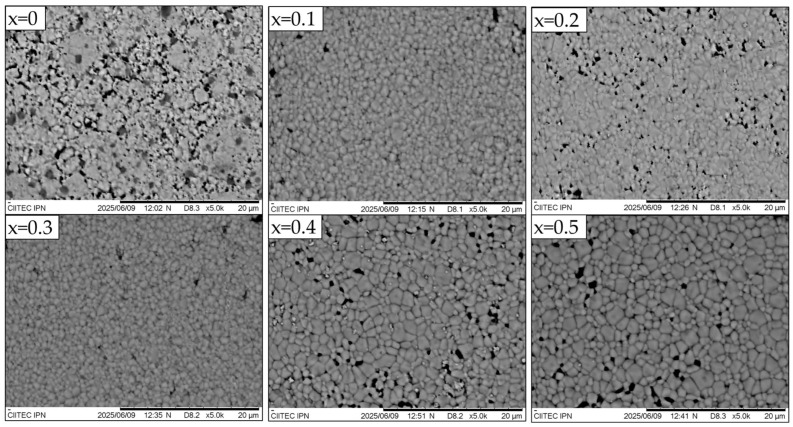
SEM micrographs of the surface of sintered pellets obtained from samples prepared by mixing La_2_O_3_, Fe_2_O_3,_ and SrO powders at stoichiometric ratios, milling for 5 h, pressing, and sintering at 1473 K for 4 h for obtaining La_1−x_Sr_x_FeO_3_, with 0 ≤ x ≤ 0.5, Δx = 0.1.

Figure 3 shows SEM micrographs of the surface of sintered La_1−x_Sr_x_FeO_3_ pellets with 0 ≤ x ≤ 0.5 (Δx = 0.1). As can be seen, the undoped LaFeO_3_ (x = 0) exhibits a homogeneous distribution of polygonal and equiaxed fine grains with significant surface porosity. When strontium is added, the equiaxed grain morphology is retained with a notable reduction in porosity. These samples show more clearly defined grain boundaries, and low porosity, without the absence of cracks or fissures. At high doping levels (x = 0.3 and 0.4), the formation of multiple phases is observed. The use of backscattered electron imaging enables clear phase contrast, revealing two distinct regions: a bright phase composed of irregularly shaped small grains embedded within a light-gray matrix of equiaxed grains. This microstructural evidence is consistent with the phase segregation identified by XRD analysis. Finally, the highest strontium content (x = 0.5) shows a single phase composed of equiaxed grains. As can be observed in the same Figure 3, the undoped sample (x = 0) exhibits the smallest average diameter, approximately 0.70 μm. In contrast, the strontium-doped samples display a larger average grain size, ranging from 0.90 to 1.50 μm, depending on the strontium content.

**Figure 4:**
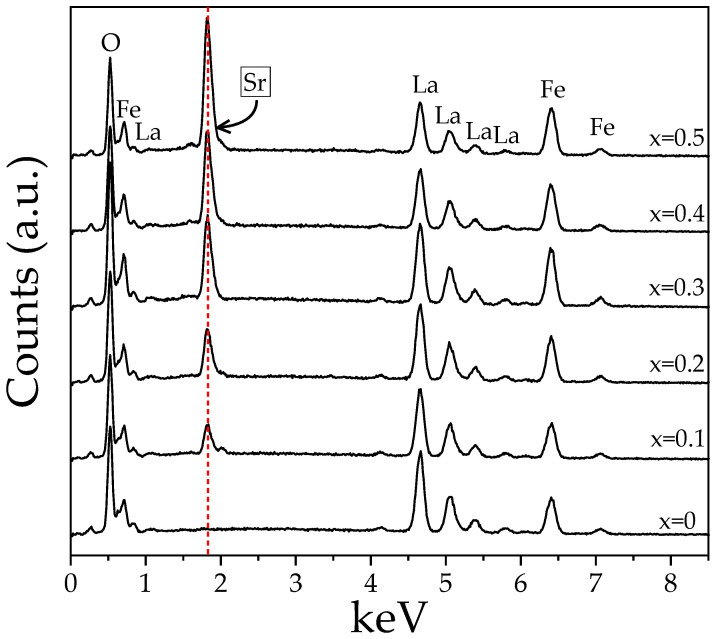
EDS spectra from SEM analyses of sintered pellets obtained from samples prepared by mixing La_2_O_3_, Fe_2_O_3,_ and SrO powders at stoichiometric ratios, milling for 5 h, pressing, and sintering at 1473 K for 4 h for obtaining La_1−x_Sr_x_FeO_3_ with 0 ≤ x ≤ 0.5, Δx = 0.1.

To provide some insight into the presence of strontium in the sintered pellets, in Figure 4, the Energy Dispersive Spectroscopy (EDS) spectra of the studied samples are presented. As can be observed, the results prove the presence of strontium in the doped samples, and also is clear the consistent increase in the strontium with the increase in x value, is in agreement with the composition analyzed.

Given that EDS is a semi-quantitative analytical technique, Table 2 shows the elemental composition obtained for the samples, which closely matches the theoretical composition expected from the synthesis process. A gradual increase in strontium content is observed as the doping level increases, accompanied by a proportional decrease in lanthanum, confirming the substitution.

### 3.4. Magnetic Properties

**Figure 5:**
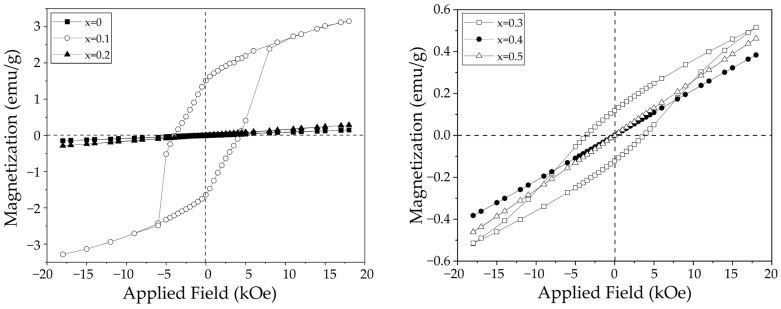
Magnetic hysteresis loops of sintered pellets obtained from samples prepared by mixing La_2_O_3_, Fe_2_O_3,_ and SrO powders at stoichiometric ratios, milling for 5 h, pressing, and sintering at 1473 K for 4 h for obtaining La_1−x_Sr_x_FeO_3_ with 0 ≤ x ≤ 0.5, Δx = 0.1.

Figure 5 displays the magnetic hysteresis loops at room temperature of sintered pellets obtained from milled mixtures of La_2_O_3_, Fe_2_O_3_, and SrO for obtaining La_1−x_Sr_x_FeO_3_ with different doping levels, x, from 0 to 0.5 mol of strontium. As it is expected, the undoped lanthanum ferrite shows antiferromagnetic behavior, consistent with a dominant antiferromagnetic (AFM) super-exchange (SE) interaction [30], exhibiting a mass magnetic susceptibility of 1.047 × 10^−10^ m^3^/kg and a specific magnetization of 0.14 emu/g at 18 kOe. When 0.1 mol of strontium is added to the pure LaFeO_3_, a weak ferromagnetic behavior is evidenced, with a specific magnetization of 3.15 emu/g at 18 kOe. This modification in the magnetic behavior is attributed to the structural distortions produced by the substitution of lanthanum for strontium, which disrupts the AFM SE interaction, responsible for modifying the magnetic structure of lanthanum ferrite.

The presence of small quantities of strontium occupying lanthanum sites prevents the cancellation of iron magnetic moments, resulting in ferromagnetism and magnetic anisotropy [31], evidenced by an increase in the coercivity and remanence, with respect to the undoped sample, achieving a value of 3.93 kOe and 1.51 emu/g, respectively. In contrast, when the strontium concentration is 0.2 mol, the magnetic behavior turns out to be antiferromagnetic, this is attributed to the fact that strontium promotes a structural distortion due to the difference in ionic radii between Sr^2+^ and La^3+^, and to the modification of the Fe-O-Fe bond angles in the crystal structure, which possibly causes the weakening of the magnetic interactions, in this case the mass magnetic susceptibility is 0.21 × 10^−10^ m^3^/kg.

When 0.3 mol of strontium is added, the material exhibits an unexpectedly weak ferromagnetic order, achieving 0.51 emu/g of specific magnetization at 18 kOe, in agreement with other studies [32]. This change in the magnetic order is accompanied by an increase in the coercivity (3.79 kOe) and remanence (0.11 emu/g), ascribed to an increased magnetocrystalline anisotropy caused by crystal lattice distortion due to strontium substitution. In addition, the coexistence of different phases generates interfaces that could act as energetic barriers, hindering the movement of domain walls and thus contributing to the increase in coercivity. For higher strontium contents, 0.4 mol and 0.5 mol of strontium, the magnetization returns to its initial AFM behavior, attributed to the AFM behavior of each phase present at both compositions.

As can be observed in Table 3, which summarizes the data obtained from the magnetic hysteresis loops of the sintered pellets with varying levels of strontium added, only the 0.1 and 0.3 mol of strontium show ferromagnetic order, attributed to the structural distortions caused by the Sr^2+^ substituting La^3+^ sites, which depends on the quantity of strontium added, as well as to the difference in magnetic moment between Fe^3+^ and Fe^4+^ species, which are created as a compensation mechanism, as previously was discussed [33].

**Figure 6:**
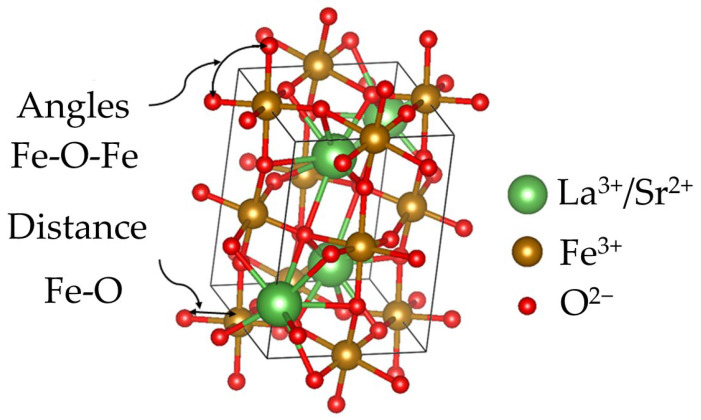
VESTA simulation of unit cell of Strontium doped LaFeO_3_ for determining the bond angles and bond distances between Fe-O of sintered pellets obtained from samples prepared by mixing La_2_O_3_, Fe_2_O_3_ and SrO powders at stoichiometric ratios, milling for 5 h, pressing and sintering at 1473 K for 4 h for obtaining La_1−x_Sr_x_FeO_3_ with 0 ≤ x ≤ 0.5, Δx = 0.1.

For a better understanding of these findings, simulations using the experimental results from XRD patterns and Rietveld refinement analysis were conducted using VESTA software version 3.5.7. Figure 6 shows the structural simulation corresponding to LaFeO_3_, where the Fe-O-Fe angles and Fe-O distances are depicted. The simulations are based on crystallographic parameters extracted from the experimental analysis, which allows for an accurate representation of the three-dimensional structure of La_1−x_Sr_x_FeO_3_ at different levels of strontium doping. In particular, changes in the Fe-O-Fe angles and Fe-O distances are detailed as the Sr content increases. The simulations not only depict the structure but also confirm the interpretation of the structural changes caused by doping, which significantly affect the physical properties of the material. It is important to remark that the magnetic behavior of bulk LaFeO_3_ is governed primarily by octahedral FeO_6_ interactions, where each Fe^3+^ ion is surrounded by six O^2−^ ions, facilitating superexchange interactions (SE) through Fe-O-Fe bonds at the shared octahedral corners. Consequently, any modification in the Fe-O-Fe bond angle directly influences the material’s magnetic ordering.

Although the Fe-O-Fe bond angle in the undoped sample is less than 180°, as is appreciated in Table 4 where the data obtained from VESTA analysis are included, it shows AFM order, which is attributed to the dominant SE interaction in this composition, due to the presence of Fe^3+^ ions located at the vertices of the crystalline lattice. When the strontium added concentration is 0.1 mol, it induces a distortion in the crystalline structure and promotes the formation of Fe^4+^, thereby inducing ferromagnetism. On the other hand, for concentrations of 0.2, 0.3, and 0.4 mol, the reported angle is 180°, consistent with AFM behavior, corroborating the magnetic behavior presented above and in accordance with the literature reports [34] for the compositions of 0.3 mol of strontium. Finally, the increase in magnetic susceptibility from 0.4 to 0.5 mol is attributed to the rhombohedral phase of La_0.6_Sr_0.4_FeO_3_, which has an angle of 174.44°, justifying the observed increase [35]. On the other hand, the decrease in Fe-O bond distances reported is consistent with those reported in the literature, which is associated with the increase in strontium concentration [36]. All the above is consistent with the results observed in X-ray diffraction and vibrating sample magnetometry.

### 3.5. Electric Properties

**Figure 7:**
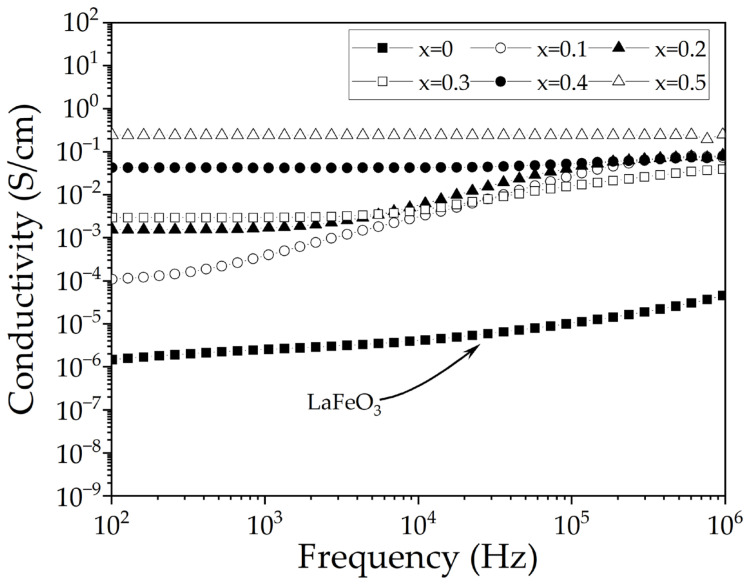
Frequency-dependent electrical conductivity of sintered pellets obtained from samples prepared by mixing La_2_O_3_, Fe_2_O_3_, and SrO powders at stoichiometric ratios, milling for 5 h, pressing, and sintering at 1473 K for 4 h for obtaining La_1−x_Sr_x_FeO_3_ with 0 ≤ x ≤ 0.5, Δx = 0.1.

In Figure 7, the electrical conductivity versus frequency, in the range of 10^2^ to 10^6^ Hz, is presented. As is observed, the conductivity increases as the dopant concentration increases, from 10^−6^ to 10^−1^ S/cm for strontium contents of 0 and 0.5 mol, respectively. It suggests that strontium generates vacancies and Fe^4+^ species to maintain electronic neutrality, as is concluded in a similar study [37]. As the frequency and the strontium concentration increase, electrical conductivity also rises, due to the presence of Fe^3+^ and Fe^4+^ species, which favor the hopping conduction mechanism, and when the concentration of Sr^2+^ increases. However, this phenomenon is mitigated by distortions in the crystal structure for x = 0.2 mol. In addition, for x = 0.3 and 0.4 mol, the presence of more than one phase and the higher number of vacancies favor the conduction mechanism. Finally, when the concentration is 0.5 mol of strontium, the structural transition to a rhombohedral phase results in increased electrical conductivity, behaving more like a conductor than a dielectric. From this, it can be inferred that conductivity is influenced by factors such as crystal structure and the presence of metastable phases, promoting greater electrical conductivity. Consequently, lanthanum-strontium ferrite exhibits electrical behavior analogous to an ionic conductor as strontium content increases [38].

### 3.6. Dielectric Properties

**Figure 8:**
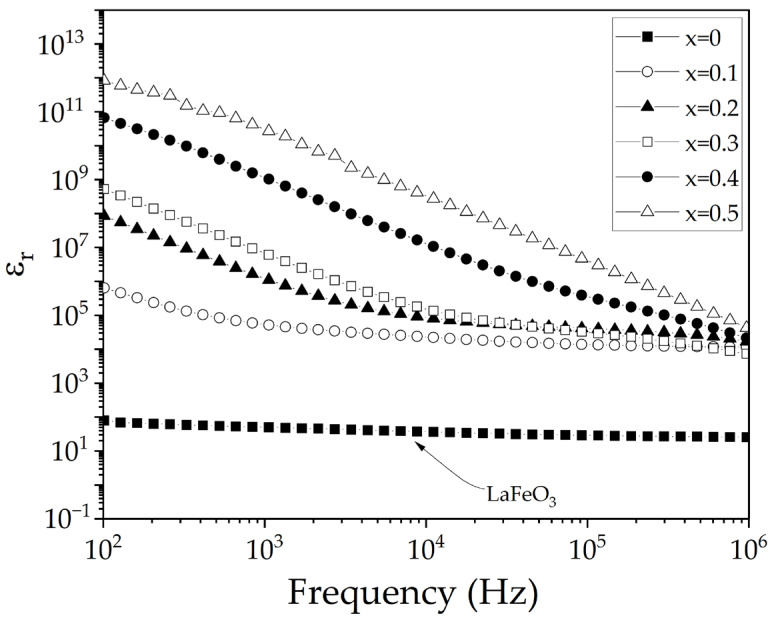
Frequency-dependent relative permittivity of sintered pellets obtained from samples prepared by mixing La_2_O_3_, Fe_2_O_3,_ and SrO powders at stoichiometric ratios, milling for 5 h, pressing, and sintering at 1473 K for 4 h for obtaining La_1−x_Sr_x_FeO_3_ with 0 ≤ x ≤ 0.5, Δx = 0.1.

The study of electrical and dielectric properties is fundamental, since knowing characteristics such as electrical conductivity, permittivity, and dielectric losses, among others, allows understanding the behavior of the material and broadening its application potential in devices that require unique and specialized properties. In Figure 8, the frequency-dependent relative permittivity, ε_r_, for all compositions is depicted. The results show an increase in permittivity as the strontium concentration in the lanthanum ferrite increases. At low frequencies (10^2^ Hz), relative permittivity values range from 10^2^ to 10^12^ for 0 and 0.5 mol of strontium, attributed to different polarization mechanisms including electron, ion, dipole motion, and grain boundary effects [39]. The substitution of La^3+^ by Sr^2+^ induces oxygen vacancies and Fe^4+^ species formation [40], attributing their formation to the mechanism of charge neutrality maintenance, in addition to the presence of different phases, porosity, and grain boundaries observed by SEM, factors that contribute to the increase in relative permittivity via the Maxwell–Wagner effect. Conversely, ε_r_ decreases with increasing frequency, resulting in values around 10 for 0 mol of strontium (undoped sample) and around 10^4^ for the other compositions, at high frequencies (10^6^ Hz), values related to the generation of vacancies and different iron species [41].

**Figure 9:**
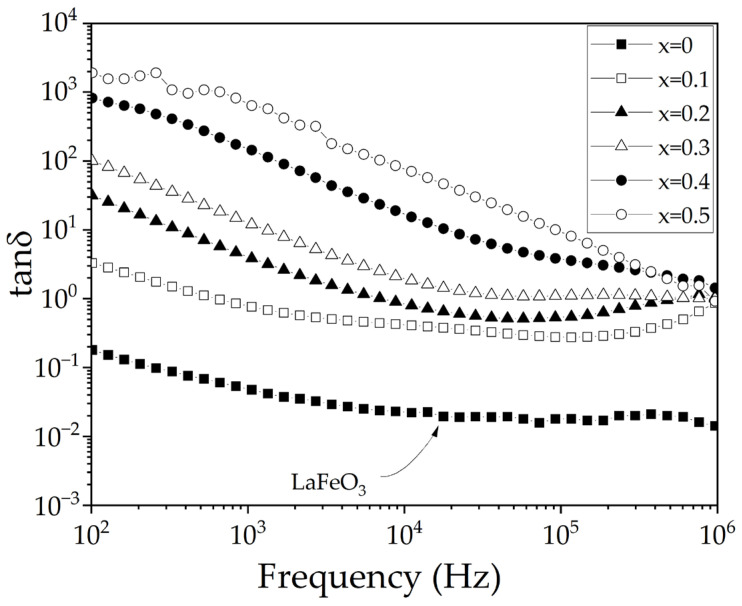
Frequency-dependent tanδ of sintered pellets, at room temperature, obtained from samples prepared by mixing La_2_O_3_, Fe_2_O_3,_ and SrO powders at stoichiometric ratios, milling for 5 h, pressing, and sintering at 1473 K for 4 h for obtaining La_1−x_Sr_x_FeO_3_ with 0 ≤ x ≤ 0.5, Δx = 0.1.

Figure 9 illustrates the frequency-dependent variation in the dielectric loss tangent, tanδ, at room temperature in the range from 10^2^ to 10^6^ Hz. As can be seen, for all the cases analyzed, the higher the strontium content, the higher the value of tanδ. At low frequencies (10^2^ Hz), a progressive increment of tanδ, attributed to the Maxwell–Wagner effect, to the vacancies generated by the charge compensation mechanisms, and to the different oxidation states of the iron species formed. Conversely, at higher frequencies, the dielectric loss tangent gradually decreases, converging to a value around 1 at a frequency of 10^6^ Hz, for all samples containing strontium.

**Figure 10:**
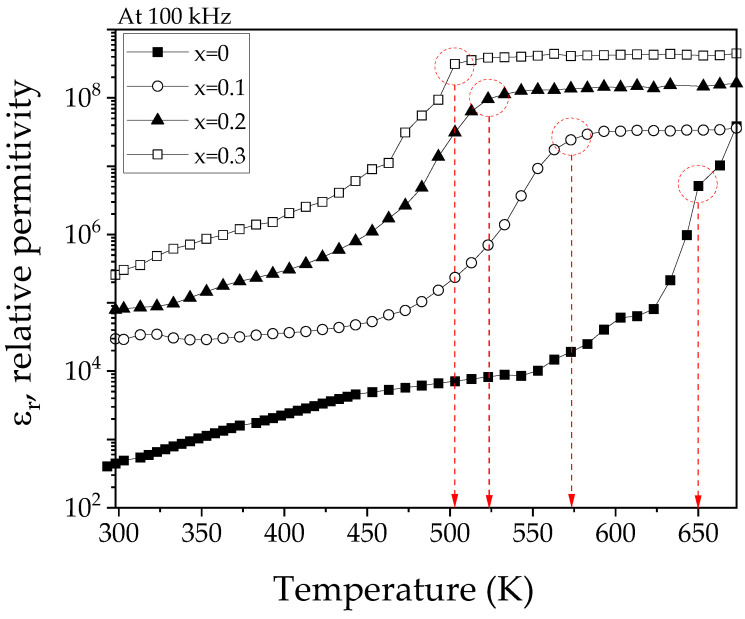
Dependence of the relative permittivity with temperature of sintered pellets obtained from samples prepared by mixing La_2_O_3_, Fe_2_O_3,_ and SrO powders at stoichiometric ratios, milling for 5 h, pressing, and sintering at 1473 K for 4 h for obtaining La_1−x_Sr_x_FeO_3_ with 0 ≤ x ≤ 0.5, Δx = 0.1.

To evaluate the effect of strontium doping on the relative permittivity, Figure 10 presents the curves obtained at 100 KHz for sintered La_1−x_Sr_x_FeO_3_ pellets, with x ranging from 0 to 0.3 mol of strontium, in the temperature range from 298 K to 675 K. As expected, all samples show a gradual increase in relative permittivity with increasing temperature until a point is reached where the curves show a plateau behavior. Beyond this point, permittivity remains constant, which is attributed to the transition from the ferroelectric to the paraelectric state. It is important to highlight that the temperature transition decreases from 650 K to 510 K as the strontium concentration increases from 0 to 0.3 mol. This reduction is ascribed to the decrease in the system’s tetragonality due to Sr^2+^ substitution, which weakens the intrinsic dipole moment and consequently lowers the transition temperature. At a frequency of 100 kHz, the dominant polarization mechanism in ferroelectric materials such as La_1−x_Sr_x_FeO_3_ is ionic or displacement polarization. This mechanism involves the cooperative displacement of ions within the crystal structure, resulting in spontaneous polarization. This behavior is characteristic of displacement-type ferroelectric materials, where polarization arises from structural changes in the crystal lattice.

### 3.7. Magnetodielectric Coupling

Finally, the magnetodielectric coupling, which quantifies the influence of an external magnetic field on the dielectric response, particularly the relative permittivity, was employed to assess the interaction between ferromagnetism and ferroelectricity. The magnetodielectric coupling (*MD*) can be indirectly determined by measuring variations in the relative permittivity under an applied magnetic field, following the expression given in (2) [24]:

(2) $$MD\left(\%\right)=100\cdot \frac{\varepsilon \left(H\right)-\varepsilon \left(0\right)}{\varepsilon \left(0\right)}=\frac{∆\varepsilon }{\varepsilon \left(0\right)}$$

where *ε*(0) and *ε*(H) are the relative permittivity when a magnetic field equal to 0 and H, respectively, is applied, and ∆*ε* is the change in relative permittivity under different magnetic fields.

**Figure 11:**
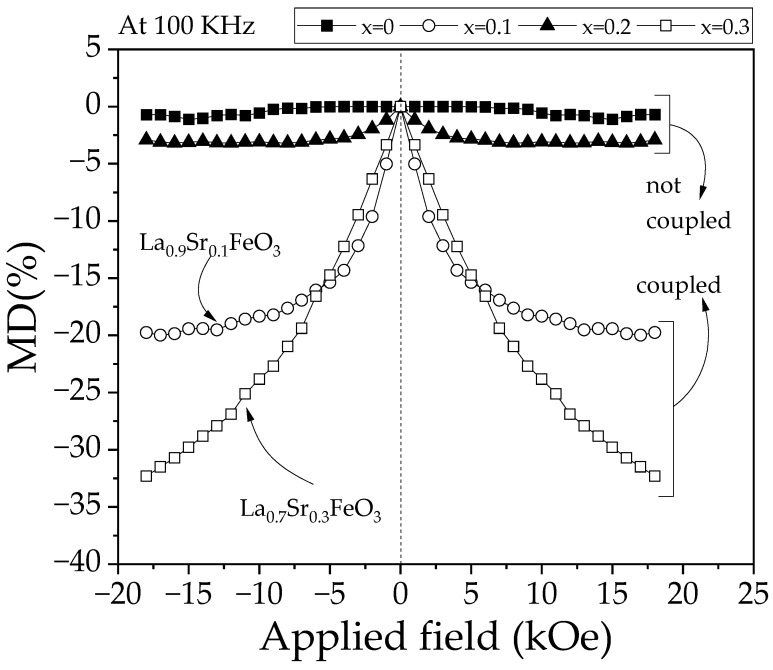
Magnetodielectric coupling (MD) of sintered pellets obtained from samples prepared by mixing La_2_O_3_, Fe_2_O_3,_ and SrO powders at stoichiometric ratios, milling for 5 h, pressing, and sintering at 1473 K for 4 h for obtaining La_1−x_Sr_x_FeO_3_ with 0 ≤ x ≤ 0.3, Δx = 0.1 at 100 kHz.

The magnetodielectric effect depends intrinsically on the properties of the material, although its magnitude and behavior are influenced by factors such as the frequency and intensity of the applied field. In some cases, the presence of different phases can favor an increase in the magnetodielectric values [42,43]. Figure 11 illustrates the variation in magnetodielectric (MD) coupling as a function of strontium concentration (x) in La_1−x_Sr_x_FeO_3_ under an applied magnetic field of ±18 kOe. For the undoped sample, no significant modifications in MD values are observed, indicating the absence of magnetodielectric coupling in this composition. As the strontium concentration increases to 0.1 mol, a noticeable enhancement in MD is evident, reaching a maximum value of approximately 20%. This behavior is attributed to the ferromagnetic nature of the dominant phase in this composition, where the interaction between ferromagnetic and ferroelectric orders is more pronounced. As was presented previously, this composition shows weak ferromagnetic order, which contributes to the coupling.

When the ferrite is doped with 0.2 mol of strontium, MD values decrease to around 3%, and the magnetic field does not affect this coupling. This reduction is associated with a transition in the ferrite’s magnetic behavior from ferromagnetic to antiferromagnetic. Additionally, structural distortions induced by the substitution of La^3+^ by Sr^2+^ may contribute to this MD response. Previous studies have reported that Sr^2+^ doping in similar materials can induce significant structural changes, influencing magnetodielectric properties [36]. Finally, for 0.3 mol of strontium, a significant increase in MD is observed, reaching a maximum of approximately 32% under applied fields of ±18 kOe. This enhancement is attributed to the coexistence of multiple phases within the composition, along with the contribution of dominant ferromagnetic behavior. Studies on related materials suggest that the presence of multiple phases can enhance magnetodielectric coupling due to complex interactions between coexisting phases [44]. The MD values observed in La_1−x_Sr_x_FeO_3_ exceed those reported in other materials, where maximum values typically reach around 10% [45].

## 4. Conclusions

Sr-doped LaFeO_3_ ceramics were successfully synthesized by high-energy ball milling followed by thermal treatment, achieving single-phase orthorhombic structures up to 0.2 mol of Sr^2+^. Phase transitions to rhombohedral and tetragonal symmetries occurred at higher doping levels. The introduction of Sr^2+^ induced weak ferromagnetism at 0.1 and 0.3 mol, associated with structural distortion and enhanced double exchange interactions. All samples exhibited high permittivity at low frequencies and a decrease in dielectric constant with increasing frequency. A transition from dielectric to semiconducting behavior was observed as the Sr content increased. The ferroelectric-paraelectric transition temperature remained above 500 K across all compositions. Notably, a magnetodielectric coupling of 32% at room temperature was obtained, demonstrating the material’s potential for multifunctional device applications.

## Figures and Tables

**Table 1:** Rietveld refinement parameters from XRD patterns of samples prepared by mixing La_2_O_3_, Fe_2_O_3,_ and SrO powders at stoichiometric ratios, milling for 5 h, pressing, and sintering at 1473 K for 4 h for obtaining La_1−x_Sr_x_FeO_3_ with 0 ≤ x ≤ 0.5, Δx = 0.1.

Sr^2+^ Content (x, mol)	Phase S.G.	Phase wt.%	Lattice Parameters (Å)			Crystallite Size (nm)	Microstrain (×10^−4^)	Goodness of Fit	
			a	b	c			R_wp_	χ^2^
0	LaFeO_3_ *Pnma*	100 ±0.0	5.55 ±4 × 10^−3^	7.86 ±4 × 10^−3^	5.55 ±5 × 10^−3^	189.34 ±2.93	5.02 ±0.23	20.29	1.08
0.1	LaFeO_3_ *Pnma*	100 ±0.0	5.53 ±9 × 10^−3^	7.84 ±8 × 10^−3^	5.55 ±9 × 10^−3^	151.98 ±3.08	5.99 ±0.17	20.44	1.06
0.2	LaFeO_3_ *Pnma*	100 ±0.0	5.52 ±3 × 10^−3^	7.81 ±5 × 10^−3^	5.55 ±3 × 10^−3^	121.54 ±1.73	2.13 ±0.71	19.59	1.11
0.3	LaFeO_3_ *Pnma*	22.65 ±0.0	5.50 ±2 × 10^−3^	7.80 ±2 × 10^−3^	5.53 ±1 × 10^−3^	122.82 ±5.52	3.72 ±1.16	17.90	1.02
	La_0.6_Sr_0.4_FeO_2.976_ *R-3c*	71.35 ±6.45	5.54 ±4 × 10^−3^	-	13.44 ±2 × 10^−3^	57.34 ±5.80	5.01 ±0.05		
	La_0.8_Sr_1.2_FeO_3.714_ *I4/mmm*	5.99 ±0.46	3.87 ±1 × 10^−3^	-	12.71 ±2 × 10^−3^	9.99 ±0.0	3.54 ±0.0		
0.4	La_0.6_Sr_0.4_FeO_2.976_ *R-3c*	90.91 ±0.0	5.52 ±3 × 10^−3^	-	13.44 ±8 × 10^−3^	98.10 ±1.46	6.04 ±0.28	18.86	1.07
	La_0.8_Sr_1.2_FeO_3.714_ *I4/mmm*	9.08 ±0.29	3.87 ±2 × 10^−3^	-	12.74 ±1 × 10^−3^	10.14 ±0.0	0.08 ±0.0		
0.5	La_0.6_Sr_0.4_FeO_2.976_ *R-3c*	100 ±0.0	5.49 ±7 × 10^−3^	-	13.51 ±3 × 10^−3^	100.02 ±1.53	6.75 ±0.76	20.79	1.08

**Table 2:** Chemical elemental composition obtained from EDS analysis of samples prepared by mixing La_2_O_3_, Fe_2_O_3,_ and SrO powders at stoichiometric ratios, milling for 5 h, pressing, and sintering at 1473 K for 4 h for obtaining La_1−x_Sr_x_FeO_3_ with 0 ≤ x ≤ 0.5, Δx = 0.1.

Sr^2+^ Content (x, mol)	Element (at%)			
	La	Sr	Fe	O
0	20.23	0	22.78	56.99
0.1	16.38	4.39	20.83	58.39
0.2	15.47	6.10	21.62	56.81
0.3	11.89	7.66	20.87	59.57
0.4	10.74	10.75	20.48	58.04
0.5	8.48	14.51	19.91	57.10

**Table 3:** Specific magnetization (M_s_), mass magnetic susceptibility (χ_m_), coercive field (H_c_), and magnetic remanence (M_r_) of the sintered pellets obtained from samples prepared by mixing La_2_O_3_, Fe_2_O_3_ and SrO powders at stoichiometric ratios, milling for 5 h, pressing and sintering at 1473 K for 4 h for obtaining La_1−x_Sr_x_FeO_3_ with 0 ≤ x ≤ 0.5, Δx = 0.1.

Sr^2+^ Content (x, mol)	M_s_ at 18 kOe (emu/g)	χ_m_ (×10^−10^ m^3^Kg^−1^)	H_c_ (kOe)	M_r_ (emu/g)	Magnetic Behavior
0	0.14	1.04 ± 0.01	-	-	AFM
0.1	3.15	-	3.93	1.51	FM
0.2	0.28	0.20 ± 0.01	-	-	AFM
0.3	0.51	-	3.79	0.11	FM
0.4	0.38	0.27 ± 0.02	-	-	AFM
0.5	0.46	0.32 ± 0.03	-	-	AFM

AFM: antiferromagnetic; FM: ferromagnetic.

**Table 4:** Bond angles and bond distances between Fe-O of sintered pellets of the sintered pellets obtained from samples prepared by mixing La_2_O_3_, Fe_2_O_3_, and SrO powders at stoichiometric ratios, milling for 5 h, pressing, and sintering at 1473 K for 4 h for obtaining La_1−x_Sr_x_FeO_3_ with 0 ≤ x ≤ 0.5, Δx = 0.1.

Sr^+2^ Content, x (mol)	Angle Fe-O-Fe (Degree)	Distance Fe-O (Å)
0	157.89	2.00
0.1	153.71	2.03
0.2	180	1.95
0.3	180	1.92
0.4	180	1.93
0.5	174.44	1.95

## Data Availability

The original contributions presented in the study are included in the article; further inquiries can be directed to the corresponding author.
